# Association of ROX Index with Mechanical Ventilator Use in Sepsis Patients in the Emergency Department

**DOI:** 10.3390/jcm11020342

**Published:** 2022-01-11

**Authors:** Sejoong Ahn, Jonghak Park, Juhyun Song, Jooyeong Kim, Hanjin Cho, Sungwoo Moon

**Affiliations:** Department of Emergency Medicine, Korea University Ansan Hospital, Ansan-si 15355, Korea; sejoongahn@naver.com (S.A.); rosc@hanmail.net (J.P.); songcap97@gmail.com (J.S.); blj01he@gmail.com (J.K.); chohj327@korea.ac.kr (H.C.)

**Keywords:** sepsis, emergency department, mechanical ventilator, ROX index

## Abstract

Detecting sepsis patients who are at a high-risk of mechanical ventilation is important in emergency departments (ED). The respiratory rate oxygenation (ROX) index is the ratio of tissue oxygen saturation/fraction of inspired oxygen to the respiratory rate. This study aimed to investigate whether the ROX index could predict mechanical ventilator use in sepsis patients in an ED. This retrospective observational study included quick sequential organ failure assessment (qSOFA) ≥ 2 sepsis patients that presented to the ED between September 2019 and April 2020. The ROX and ROX-heart rate (HR) indices were significantly lower in patients with mechanical ventilator use within 24 h than in those without the use of a mechanical ventilator (4.0 [3.2–5.4] vs. 10.0 [5.9–15.2], *p* < 0.001 and 3.9 [2.7–5.8] vs. 10.1 [5.4–16.3], *p* < 0.001, respectively). The area under the receiver operating characteristic (ROC) curve of the ROX and ROX-HR indices were 0.854 and 0.816 (both *p* < 0.001). The ROX and ROX-HR indices were independently associated with mechanical ventilator use within 24 h (adjusted hazard ratio = 0.78, 95% CI: 0.68–0.90, *p* < 0.001 and adjusted hazard ratio = 0.87, 95% CI 0.79–0.96, *p* = 0.004, respectively). The 28-day mortality was higher in the low ROX and low ROX-HR groups. The ROX and ROX-HR indices were associated with mechanical ventilator use within 24 h in qSOFA ≥ 2 patients in the ED.

## 1. Introduction

Sepsis is a life-threatening condition that results from a dysregulated host response to infections [1]. Sepsis is one of the most common causes of mechanical ventilator use in intensive care units (ICUs) [2] and emergency departments (EDs) [3,4,5]. Mechanical ventilation is a key component in managing patients with intubated sepsis [6,7,8].

The respiratory rate oxygenation (ROX) index is the ratio of tissue oxygen saturation (SpO_2_)/fraction of inspired oxygen (FiO_2_) to the respiratory rate (RR) [9]. Previous studies have reported that the ROX index can be used to predict the need for mechanical ventilator use in patients with hypoxemic acute respiratory failure using a high-flow nasal cannula in ICUs [9,10]. The ROX-heart rate (HR) index is a modified ROX index that includes the heart rate and ROX index. A previous study used the ROX-HR index to identify mechanical ventilator use in patients with hypoxemic acute respiratory failure using a high-flow nasal cannula in the ICU [11].

The usefulness of the ROX index in EDs remains unclear. A previous study reported that a low ROX index in the ED could predict mortality in sepsis patients [12]. Identifying patients who are at a high-risk of mechanical ventilation is important for appropriate management. The role of the ROX or ROX-HR index in predicting the need for mechanical ventilator use regardless of the use of a high-flow nasal cannula in sepsis patients in EDs is not well studied. Thus, this study aimed to investigate whether the ROX index or ROX-HR index could predict mechanical ventilator use in quick sequential organ failure assessment (qSOFA) ≥ 2 sepsis patients in an ED.

## 2. Materials and Methods

### 2.1. Study Design and Setting

This retrospective observational study was performed at the Korea University Ansan Hospital, the only tertiary academic teaching hospital in Ansan-si. Approximately 50,000 patients visit the ED of Korea University Ansan Hospital each year [13]. This study was approved by the institutional review board (IRB) of Korea University (2021AS0057). Due to its retrospective design, the requirement for informed consent was waived by the IRB.

### 2.2. Definitions

The qSOFA score was used for screening sepsis and considered whether the patient’s RR ≥ 22/min, whether the patient’s Glasgow Coma Scale (GCS) score < 15, and whether the patient’s systolic blood pressure ≤ 100 mmHg [1]. The qSOFA score was considered “qSOFA-positive” if two or more components were positive. The sequential organ failure assessment (SOFA) score was used to diagnose sepsis and composed of six categories (respiration, coagulation, liver, cardiovascular, central nervous system, and renal system evaluations) with scores ranging from 0 to 4 for each category. An increase of two or more SOFA scores from the patient’s baseline or qSOFA positive due to infection was defined as sepsis [1]. Septic shock was defined as a lactic acid level ≥ 2 mmol/L and vasopressor use to maintain a mean arterial pressure ≥ 65 mmHg despite adequate fluid resuscitation [1]. All patients were managed according to the Surviving Sepsis Campaign guidelines [1,6].

Whether the patient was diagnosed as sepsis or not and primary infection focus was determined by the board-certified emergency physicians or infection specialist by reviewing all medical records and investigations.

The ROX index was defined as the ratio of tissue oxygen saturation (SpO_2_)/fraction of inspired oxygen (FiO_2_) to RR [9]. ROX-HR was defined as the ROX index over HR/100 [11].

ROX index = (SpO_2_)/(FiO_2_ × RR) (1)

ROX-HR index = (ROX index × 100)/HR(2)

FiO_2_ was calculated according to the methods of oxygen delivery [14]. A high concentration reservoir mask was used to supply oxygen when oxygen was delivered at flow rate of 10–15 L/min. The calculated FiO_2_ of patients using high concentration reservoir mask was ranged from 0.60 to 0.90. A simple face mask was used to supply oxygen when oxygen was delivered at flow rate of 5–10 L/min. The calculated FiO_2_ of patients using simple face mask was ranged from 0.40 to 0.60. A nasal prong was used to supply oxygen when oxygen was delivered at flow rate of 1–5 L/min. The calculated FiO_2_ of patients using nasal prong was ranged from 0.24 to 0.40. We assumed that there are linear correlations between calculated FiO_2_ and delivered flow rate of oxygen for reservoir mask, simple mask, and nasal prong. When physicians decided to use a venturi mask or high-flow nasal cannula to deliver oxygen, FiO_2_ was defined as targeted oxygen concentration by venturi mask or high-flow nasal cannula [14].

Initial values after arrival at the ED were used to calculate the qSOFA score, SOFA score, ROX index, and ROX-HR index.

The mechanical ventilator use was defined as invasive mechanical ventilator use.

### 2.3. Study Population

From September 2019 to April 2020, adult patients (age ≥ 18 years) that were qSOFA-positive upon ED arrival and were diagnosed with sepsis were included. Patients who were under 18 years old, pregnant, arrived in cardiac arrest, had missing laboratory results needed to calculate the SOFA score or ROX index, had a do-not-resuscitate (DNR) order for mechanical ventilator or were not willing to be resuscitated with mechanical ventilator, or were in an intubated state or tracheostomy state before arrival to the ED were excluded. Patients who did not survive for more than 24 h were excluded as this might have affected the primary outcome.

### 2.4. Data Collection

Patient data were obtained from the electronic medical records. Baseline characteristics of the patients, such as sex, age, and comorbidities, were collected. The patient’s initial vital signs upon arrival to the ED, infection focus, laboratory results department, SOFA scores, vasopressor use, and septic shock status in the ED were collected.

The comorbidities of patients were classified as heart disease, lung disease, liver disease, kidney disease, cerebrovascular disease, and malignancy if they were diagnosed with congestive heart failure, prior myocardial infarction, or cardiomyopathy; chronic obstructive pulmonary disease or interstitial primary fibrosis; liver cirrhosis; chronic kidney disease; hemorrhagic or ischemic stroke; and active malignancy, respectively.

The infection focus was categorized into respiratory, central nervous system, gastrointestinal, genitourinary, skin and soft tissue, catheter-related, and bacteremia. Brain Natriuretic Peptide (BNP) elevation was defined as an elevation in the N-termial pro-BNP (NT-proBNP) or BNP levels.

### 2.5. Outcomes

The primary outcome was the use of mechanical ventilator within 24 h after ED arrival. The secondary outcomes were 7-day mortality, 14-day mortality, 28-day mortality, and ICU admission.

### 2.6. Statistical Analysis

Continuous variables were expressed as means and standard deviations when the variables were normally distributed or medians and interquartile ranges when the variables were not normally distributed. Continuous variables were compared using Student’s *t*-test or the Mann–Whitney test according to the distribution of variables. Categorical variables were expressed as numbers and percentages and were evaluated using the chi-square test or Fisher’s exact test. The receiver operating characteristic (ROC) curve of the ROX index and ROX-HR index predicting the primary outcome was performed to determine the area under the curve, sensitivity, and specificity. The Youden index was used to determine the optimal cutoff point. The primary outcome and secondary outcomes by ROX and ROX-HR indices levels according to the cutoff points were evaluated. When evaluating associations of 7-day, 14-day, and 28-day mortality with ROX and ROX-HR indices levels according to the cutoff points, the patients who died within 24 h were included in analysis to reduce selection bias. Kaplan–Meier analysis and the log-rank test were performed to compare mechanical ventilator use within 24 h by ROX and ROX-HR indices levels according to the cutoff points. The Cox proportional hazard model was performed to find factors associated with mechanical ventilator use within 24 h. The factors that were significant at a level of 0.1 and those selected by the researchers were used in a stepwise backward elimination multivariate Cox proportional hazard model (Supplementary Table S1). Subgroup analysis according to infection focus was performed. A *p*-value of less than 0.05 was considered significant. Statistical analyses were performed using R version 4.0.2 (R Foundation for Statistical Computing, Vienna, Austria).

## 3. Results

**Figure 1:**
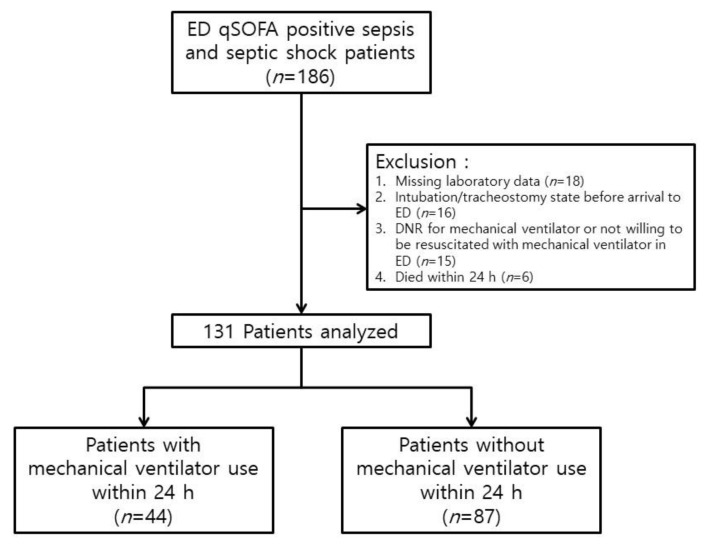
Flow chart of the study population. Abbreviations: ED = emergency department, qSOFA = quick sequential organ failure assessment, DNR = do-not-resuscitate.

During the study period, 186 patients were qSOFA-positive with a diagnosis of sepsis or septic shock. Forty-nine patients were excluded due to: missing laboratory data (18 patients), intubated or tracheostomy state before arrival at the ED (16 patients), DNR order or unwillingness to be resuscitated (15 patients), and death within 24 h (6 patients). Finally, 131 patients were included (Figure 1).

### 3.1. Baseline Characteristics

Of 131 patients, the mean age was 75.0 ± 12.5, and 75 (57.3%) were male. The mean SOFA score was 8.8, and 55 (42.0%) patients were diagnosed with septic shock. Respiratory infection was the most frequent primary infection focus in the study population. None of the patients were diagnosed with COVID-19.

Table 1 shows the baseline and clinical characteristics according to the use or non-use of mechanical ventilator within 24 h. The ROX and ROX-HR indices were significantly lower in patients with mechanical ventilator use within 24 h than in patients without the use of a mechanical ventilator (4.0 [3.2–5.4] vs. 10.0 [5.9–15.2], *p* < 0.001 and 3.9 [2.7–5.8] vs. 10.1 [5.4–16.3], *p* < 0.001, respectively). SpO_2_, pH, PO_2_, and PaO_2_/FiO_2_ ratio were significantly lower in patients with mechanical ventilator use within 24 h than in those without. The SOFA score, SOFA without respiration score, septic shock status, RR, lactate level, PCO_2_, vasopressor use, and applied FiO_2_ were statistically higher in patients with mechanical ventilator use within 24 h than in those without. There was no statistical difference in the use of a high-flow nasal cannula between the two groups.

### 3.2. ROC Curve of ROX Index and ROX-HR Index for Mechanical Ventilator Use within 24 h

**Figure 2:**
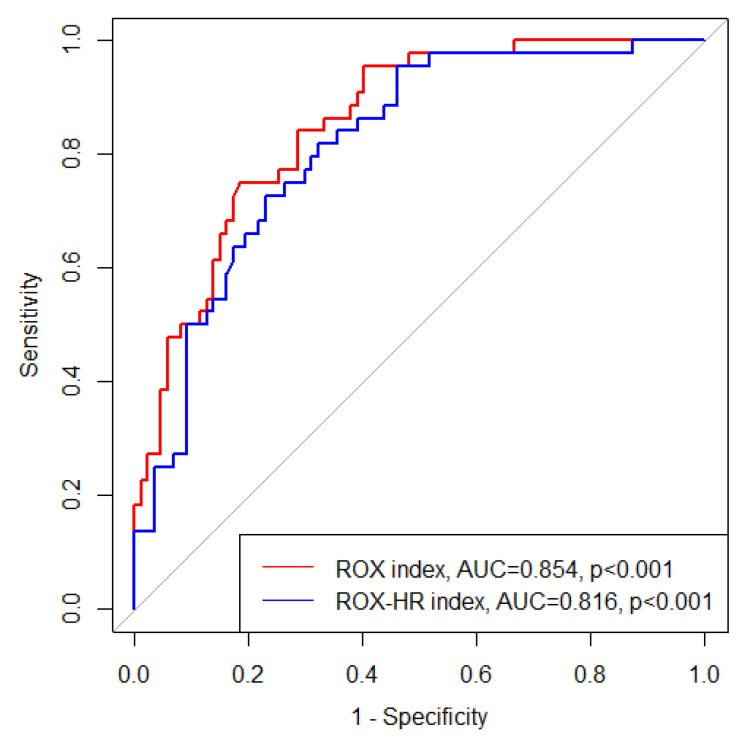
Receiver operating characteristic curve of the ROX index and ROX-HR index for mechanical ventilator use within 24 h. Abbreviations: ROX = respiratory rate oxygenation, AUC = area under curve.

The area under the ROC (AUROC) curve of the ROX index for mechanical ventilator use within 24 h was 0.854 (95% CI: 0.791–0.918, *p* < 0.001) (Figure 2). The optimal cutoff point was 5.238, with a sensitivity of 75.0% and specificity of 81.6%. The AUROC curve of ROX-HR was 0.816 (95% CI: 0.742–0.890, *p* < 0.001). The optimal cutoff point was 5.210, with a sensitivity of 72.7% and specificity of 77.0%.

### 3.3. Outcomes according to the ROX Index and ROX-HR Index Level

When the ROX index was grouped according to the optimal cutoff from the ROC curve, the use of mechanical ventilator within 24 h was significantly higher in the low ROX group than in the high ROX group (*p* < 0.001). The 7-day mortality, 14-day mortality, 28-day mortality, and ICU admissions were also significantly higher in the low ROX group than in the high ROX group (*p* < 0.05) (Table 2).

When grouping the ROX-HR index by the cutoff point, the primary outcome was significantly higher in the low ROX-HR group than in the high ROX-HR group (*p* < 0.001). Among the secondary outcomes, 7-day mortality, 14-day mortality, 28-day mortality and ICU admission were significantly higher in the low ROX group than in the high ROX group (*p* < 0.05) (Table 2).

### 3.4. Multivariate Cox Proportional Hazard Model

**Figure 3:**
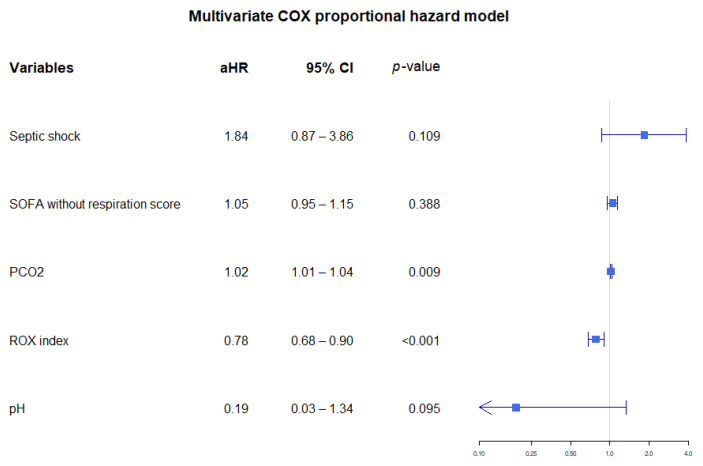
Multivariate cox proportional hazard model using the ROX index. Abbreviations: aHR = adjusted hazard ratio, CI = confidence interval, SOFA = sequential organ failure assessment, PCO_2_ = partial pressure of carbon dioxide.

In the multivariate Cox proportional hazard model using ROX index, ROX index was independently associated with mechanical ventilator use within 24 h (adjusted hazard ratio = 0.78, 95% CI: 0.68–0.90, *p* < 0.001), (Figure 3).

**Figure 4:**
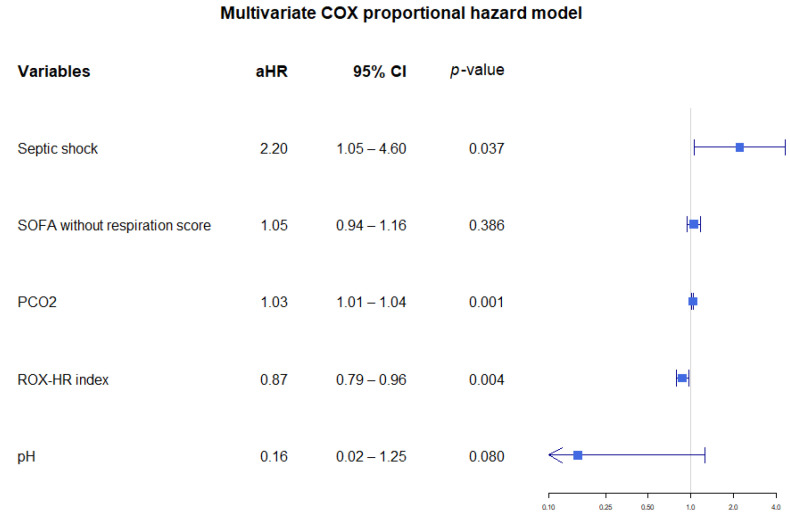
Multivariate cox proportional hazard model using the ROX-HR index. Abbreviations: aHR = adjusted hazard ratio, CI = confidence interval, SOFA = sequential organ failure assessment, PCO_2_ = partial pressure of carbon dioxide.

In the multivariate Cox proportional hazard model using ROX-HR index, ROX-HR index was independently associated with mechanical ventilator use within 24 h (adjusted hazard ratio = 0.87, 95% CI 0.79–0.96, *p* = 0.004), (Figure 4).

### 3.5. Kaplan-Meier Curve

**Figure 5:**
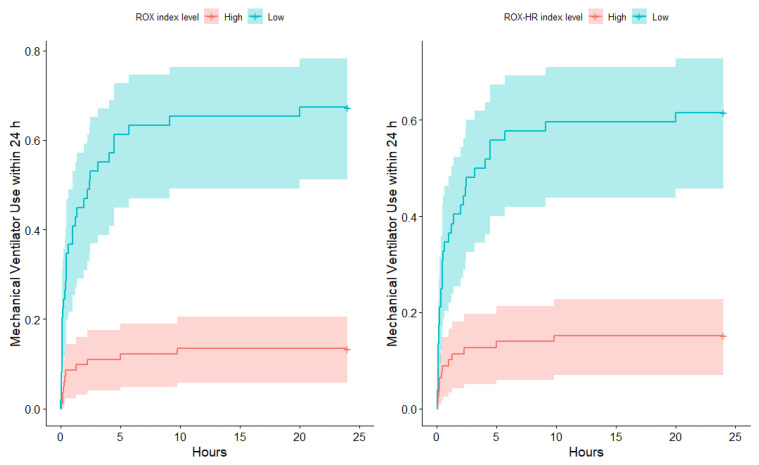
Kaplan–Meier curve for mechanical ventilator use within 24 h by ROX index (**left**) and ROX-HR index (**right**) level.

Figure 5 shows that mechanical ventilator use within 24 h was higher in low ROX index and low ROX-HR groups (log-rank test: both *p* < 0.001).

### 3.6. Subgroup Analysis

The primary origin of infection focus of 87 patients was respiratory cause. Among them, ROX and ROX-HR indices were both lower in patients with mechanical ventilator use within 24 h than in patients without the use of a mechanical ventilator (3.6 [2.9–4.5] vs. 8.9 [5.5–13.5], *p* < 0.001 and 3.6 [2.5–6.3] vs. 7.3 [4.9–13.5], *p* < 0.001, respectively). The AUROC curve of ROX index and ROX-HR were 0.844 (95% CI: 0.760–0.927, *p* < 0.001) and 0.778 (95% CI: 0.679–0.877, *p* < 0.001), respectively. ROX index was independently associated with mechanical ventilator use within 24 h (adjusted hazard ratio = 0.81, 95% CI: 0.68–0.95, *p* = 0.012), while ROX-HR was not (adjusted hazard ratio = 0.91, 95% CI: 0.82–1.01, *p* = 0.071).

The primary origin of infection focus of 44 patients was other than respiratory cause. Among them, ROX and ROX-HR indices were both lower in patients with mechanical ventilator use within 24 h than in patients without the use of a mechanical ventilator (4.9 [4.2–6.2] vs. 11.7 [7.4–19.8], *p* < 0.001 and 4.3 [3.3–5.7] vs. 12.9 [6.5–23.9], *p* < 0.001, respectively). The AUROC curve of ROX index and ROX-HR were 0.856 (95% CI: 0.747–0.965, *p* < 0.001) and 0.882 (95% CI: 0.782–0.982, *p* < 0.001), respectively. ROX index was independently associated with primary outcome (adjusted hazard ratio = 0.71, 95% CI 0.51–0.99, *p* = 0.049), while ROX-HR was not (adjusted hazard ratio = 0.65, 95% CI 0.37–1.13, *p* = 0.126).

## 4. Discussion

The ROX and ROX-HR indices in qSOFA-positive sepsis patients in the ED were independently associated with mechanical ventilator use within 24 h. The AUROC curves of the ROX and ROX-HR indices for mechanical ventilator use within 24 h showed good values (0.854 and 0.816, respectively). According to the optimal cutoff, the low ROX and low ROX-HR groups were also associated with short-term mortality.

Critical patients on mechanical ventilation require considerable medical resources [15]. In sepsis patients, mechanical ventilation is also a component of the respiratory evaluation of the SOFA score, which is associated with mortality [6]. It is important to identify patients at a high-risk of mechanical ventilator use in emergency settings.

The PaO_2_/FiO_2_ ratio is traditionally used to assess oxygenation in patients with acute respiratory failure, especially for diagnosing acute lung injury or acute respiratory distress syndrome [16]. Obtaining PaO_2_ from patients requires arterial blood sampling. SpO_2_ can be obtained non-invasively using pulse oximetry and correlates well with PaO_2_ in the range of 80–100% [17]. Rice et al. revealed that the SpO_2_/FiO_2_ ratio correlated with the PaO_2_/FiO_2_ ratio in patients with acute lung injury or acute respiratory failure (r = 0.89, *p* < 0.001) [18]. Roca et al. studied the association of indexes with high-flow nasal cannula failure, defined as mechanical ventilator use in patients with acute respiratory failure in the ICU [9,10]. They combined the SpO_2_/FiO_2_ ratio with RR, which showed an association with mechanical ventilator use. They named the ratio of SpO_2_/FiO_2_ to RR as the ROX (respiratory rate oxygenation) index. Goh et al. then introduced the ROX-HR index by adding HR with the ROX index [11]. Both ROX and ROX-HR indices can be obtained easily and non-invasively, especially in triage or at the bedside.

The ROX index is useful for identifying patients at risk of mechanical ventilation in critical settings. Two studies by Roca et al. have reported that the ROX index can be used to predict the need for mechanical ventilator use in patients with hypoxemic acute respiratory failure using a high-flow nasal cannula in ICUs [9,10]. Our study showed a higher AUROC curve than previous studies (0.740 and 0.752) using the initially measured ROX index. The optimal cutoff point was similar to that reported previously. Compared to previous studies showing independent association between mechanical ventilator use and ROX index as categorical variable, we found an independent association of ROX index with use of a mechanical ventilator as a continuous variable (adjusted hazard ratio = 0.78, 95% CI: 0.68–0.90, *p* < 0.001). The inclusion criteria of initial qSOFA-positive patients might have contributed to the higher AUROC curve, even though the initial ROX index was considered. It is also possible that patients in the ICU received initial resuscitation in the ED or general wards before admission to ICU, which may contribute to the difference in the AUROC curve. The ROX index might be a more promising tool for identifying high-risk patients in an ED than in ICU.

The ROX-HR index is a modified ROX index that adds HR to the ROX index. Goh et al. have reported ROX-HR index as a better tool for identifying mechanical ventilator use in acute hypoxemic failure patients with high-flow nasal cannula [11]. However, our study showed a greater AUROC curve for the ROX index than the ROX-HR index (0.854 vs. 0.816, *p* = 0.020). This might be explained by the fact that the HR of our population did not differ significantly between the groups, whereas the HR was significantly higher in the group with mechanical ventilator use in a previous study. For patients who are qSOFA-positive sepsis in the ED, the ROX index might be a better tool to predict mechanical ventilator use within 24 h than ROX-HR index. Pimentel et al. reported that HR did not change significantly in critical COVID-19 patient those with low SpO_2_ and high FiO_2_ [19]. This reflects that ROX index might be a better index to predict mechanical ventilator use in critical COVID-19 patient than ROX-HR index.

In previous studies, both ROX and ROX-HR indices at delayed time points, especially at 10 and 12 h, showed a better AUROC curve [9,10,11]. Further research is warranted to determine the ROX and ROX-HR index values in the ED at different time points, such as 2, 6, 10, 12 h, and beyond.

ROX index might be a fine tool to predict mechanical ventilator use within 24 h regardless of infection focus. The AUROC curves of ROX index were similar between subgroups and whole study population. ROX index showed independent association with mechanical ventilator use in both subgroups, while ROX-HR did not. The AUROC curve of ROX-HR index was smaller in patients with primary infection of respiratory cause than whole study population. For those with respiratory infection, the changes of HR might be not significant in those who need mechanical ventilation. Therefore, ROX index might be a better index to predict mechanical ventilator use than ROX-HR index, especially in patients with respiratory infection.

The ROX and ROX-HR indices can be used as additional tools for identifying sepsis patients with a high-risk of mortality in the ED. Lee et al. reported that a ROX index of less than ten was independently associated with mortality in sepsis patients in the ED (adjusted hazard ratio 1.41) [12]. Our study showed that the low ROX index group and the low ROX-HR index group were associated with short-term mortality in sepsis patients, supporting the results of a previous study. Respiratory dysfunction can decrease the SpO_2_/FiO_2_ ratio and increase RR. Renal dysfunction and acidosis in sepsis can also increase RR due to compensation. The association of the ROX index with mortality in sepsis patients can be explained by these mechanisms. To the best of our knowledge, the association of the ROX-HR index with mortality in sepsis has not yet been reported. Further studies are warranted to determine whether ROX-HR is associated with mortality in sepsis patients.

This study had several limitations. First, the factors that can affect mechanical ventilator use might have been missed due to the retrospective observational nature of this study. Second, actual inspired FiO_2_ was not obtained in our study. True inspired FiO_2_ is influenced by minute ventilation when using reservoir mask, simple mask, and nasal prong [20]. The actual minute ventilation of the patients was not obtained and not able to be calculated in our study. Therefore, FiO_2_ is estimated by assumption of linear correlations between calculated FiO_2_ and delivered flow rate of oxygen for reservoir mask, simple mask, and nasal prong. True inspired FiO_2_ might be different from estimated FiO_2_. Third, this study was performed at a single ED. Further multicenter studies are needed to generalize these results. Fourth, initial qSOFA-positive sepsis patients were included. The result cannot be generalized to initial qSOFA-negative or delayed qSOFA-positive sepsis patients. Further studies including the ROX index and ROX-HR value in initial qSOFA-negative or delayed qSOFA-positive sepsis patients are warranted. Fifth, the initial values from arrival to the ED were used for analysis. The roles of the ROX and ROX-HR indices at 6 h, 12 h, and beyond have not been determined and require further investigation. Sixth, 18 patients were excluded due to missing laboratory data that are needed for SOFA score or ROX index calculation. Most of them were excluded due to missing initial SpO_2_. This might be due to poor sensing of SpO_2_ upon arrival at ED. One patient had missing laboratory data for SOFA score calculation. The patient’s lab was performed on outpatient department, which is few days before arrival at the ED. The patient did not have any symptoms at outpatient department. The patient refused blood chemistry lab at the ED, admitted without any lab test at ED, and died on Hospital Day 3. We decided to exclude this patient because the lab result at outpatient department might not reflect true condition upon arrival to the ED. Seventh, the patients who died within 24 h were excluded in this study. Some patients might experience cardiac arrest without return of spontaneous circulation within 24 h. These patients might receive intubation but failed to use mechanical ventilator. These patients could influence the result. However, the results were similar when the patients who died within 24 h were included in analysis.

## 5. Conclusions

The ROX and ROX-HR indices were associated with mechanical ventilator use within 24 h in qSOFA-positive patients in the ED. ROX and ROX-HR indices were also associated with short-term mortality.

## Figures and Tables

**Table 1:** Baseline and clinical characteristics of the study population.

Variables	Patients without MV Use within 24 h (*n* = 87)	Patients with MV Use within 24 h (*n* = 44)	*p*-Value
Male Female	52 (59.8) 35 (40.2)	23 (52.3) 21 (47.7)	0.527
Age (years)	78.0 [71.0–84.0]	75.5 [61.0–83.0]	0.063
SOFA score	7 [5–9]	11 [10–13]	<0.001
SOFA without respiration score	6.0 ± 2.9	7.9 ± 3.1	0.001
Septic shock	25 (28.7)	30 (68.2)	<0.001
Infection focus			0.508
Respiratory	53 (60.9)	34 (77.3)	
Genitourinary	20 (23.0)	6 (13.6)	
Gastrointestinal	10 (11.5)	3 (6.8)	
Bacteremia	2 (2.3)	1 (2.3)	
CNS	1 (1.1)	0 (0)	
Catheter-related	1 (1.1)	0 (0)	
Past medical history			
Hypertension	51 (58.6)	21 (47.7)	0.318
Diabetes	41 (47.1)	20 (45.5)	1.000
Liver disease	4 (4.6)	5 (11.4)	0.280
Heart disease	22 (25.3)	6 (13.6)	0.190
Cerebrovascular disease	19 (21.8)	10 (22.7)	1.000
Lung disease	6 (6.9)	6 (13.6)	0.346
Kidney disease	5 (5.7)	6 (13.6)	0.229
Malignancy	24 (27.6)	11 (25.0)	0.915
Vital signs			
SBP (mmHg)	99.0 [89.0–130.5]	101.5 [75.5–128.0]	0.321
DBP (mmHg)	60.0 [51.0–74.0]	57.0 [50.0–73.0]	0.277
RR (/min)	23.0 [20.0–26.0]	24.0 [22.0–28.0]	0.033
HR (/min)	102.2 ± 29.0	107.1 ± 29.1	0.367
Body temperature	37.2 ± 1.3	37.1 ± 1.3	0.688
Laboratory data			
Lactate (mmol/L)	2.5 [1.7–4.5]	4.4 [2.4–6.5]	0.005
Procalcitonin (ng/mL)	1.3 [0.5–9.0]	1.9 [0.6–15.1]	0.454
Platelet count (×10^3^/µL)	209.0 [133.5–284.5]	200.5 [113.0–294.5]	0.866
White blood cells (×10^3^/µL)	11.2 [7.8–16.8]	10.6 [7.1–17.3]	0.748
pH	7.4 [7.4–7.5]	7.3 [7.2–7.4]	<0.001
PCO_2_ (mmHg)	31.5 [27.0–39.6]	38.5 [27.4–56.2]	0.017
PO_2_ (mmHg)	76.0 [60.9–97.5]	67.5 [51.0–87.0]	0.030
HCO_3_ (mmol/L)	20.5 [17.5–24.1]	19.8 [14.4–26.1]	0.936
Glucose (mg/dL)	140.0 [107.5–174.0]	149.0 [107.5–214.5]	0.477
Creatinine (mg/dL)	1.3 [0.9–2.0]	1.5 [0.9–2.5]	0.594
Bilirubin (mg/dL)	0.6 [0.4–1.1]	0.6 [0.3–0.9]	0.434
CRP (mg/dL)	11.3 [6.1–17.6]	10.2 [3.9–18.2]	0.533
Albumin (g/dL)	3.0 ± 0.6	3.0 ± 0.9	0.942
Highly sensitive troponin T (ng/mL)	0.1 [0.0–0.1]	0.1 [0.0–0.2]	0.284
BNP elevation	40 (46.0)	22 (50.0)	0.802
Clinical data			
Vasopressor use	36 (41.4)	35 (79.5)	<0.001
SpO_2_	96.0 [92.0–98.0]	90.0 [82.0–95.0]	<0.001
Applied FiO_2_	0.4 [0.3–0.6]	1.0 [0.8–1.0]	<0.001
O_2_ supplementation	73 (83.9)	41 (93.2)	0.224
High-flow nasal cannula use	18 (20.7)	8 (18.2)	0.914
PaO_2_/FiO_2_ ratio	188.5 [97.5–355.7]	72.7 [51.8–125.6]	<0.001
ROX index	10.0 [5.9–15.2]	4.0 [3.2–5.4]	<0.001
ROX-HR index	10.1 [5.4–16.3]	3.9 [2.7–5.8]	<0.001

Values are expressed as the mean ± standard deviation, median [IQR], or number (%). Abbreviations: MV = mechanical ventilator, SOFA = sequential organ failure assessment, CNS = central nervous system, GCS = Glasgow coma scale, SpO_2_ = tissue oxygen saturation, SBP = systolic blood pressure, DBP = diastolic blood pressure, RR = respiratory rate, HR = heart rate, PCO_2_ = partial pressure of carbon dioxide, PO_2_ = partial pressure of oxygen, HCO_3_ = bicarbonate, BNP = brain natriuretic peptide, PaO_2_ = partial pressure of oxygen in alveoli, ROX = respiratory rate oxygenation, CRP = C-reactive protein, FiO_2_ = fraction of inspired oxygen.

**Table 2:** Outcomes according to the ROX index level and ROX-HR index level.

**Outcomes**	**High ROX Level**	**Low ROX Level**	***p*-Value**
MV use within 24 h ^1^	11/82 (13.4)	33/49 (67.3)	<0.001
7-day mortality ^2^	6/84 (7.1)	15/53 (28.3)	0.002
14-day mortality ^2^	12/84 (14.3)	19/53 (35.8)	0.006
28-day mortality ^2^	13/84 (15.5)	23 (43.4)	0.001
ICU admission ^1^	42/82 (51.2)	36/49 (73.5)	0.020
**Outcomes**	**High ROX-HR Level**	**Low ROX-HR Level**	***p*-Value**
MV use within 24 h ^1^	12/79 (15.2)	32/52 (61.5)	<0.001
7-day mortality ^2^	8/82 (9.8)	13/55 (23.6)	0.049
14-day mortality ^2^	13/82 (15.9)	18/55 (32.7)	0.035
28-day mortality ^2^	14/82 (17.1)	22/55 (40.0)	0.005
ICU admission ^1^	39/79 (49.4)	39/52 (75.0)	0.006

^1^ The study populations (*n* = 131) were analysed. ^2^ The patients who died within 24 h (*n* = 6) were included into the analysis (total 137 patients). Values are expressed as numbers/total number (%). Abbreviations: MV = mechanical ventilator, ICU = intensive care unit.

## Data Availability

The data presented in this study are available on request from the corresponding author. The data are not publicly available due to their containing information that could compromise the privacy of research participants.

## Supplementary Materials

The following are available online at https://www.mdpi.com/article/10.3390/jcm11020342/s1, Table S1: univariate Cox proportional hazard model.
