# Supplementary material for: Association of ROX Index with Mechanical Ventilator Use in Sepsis Patients in the Emergency Department
Source: J Clin Med. 2022 Jan 11;11(2):342. doi: 10.3390/jcm11020342 (PMC8779773; doi:10.3390/jcm11020342)
Supplement: Supplementary file 1 [file jcm-11-00342-s001.zip › jcm-1516582-supplementary.pdf]

Journal of
Clinical Medicine

Table S1. Univariate Cox proportional hazard model.

Variables	Hazard ratio	95% CI	*p*-Value
Sex, ref = female	0.77	0.42–1.38	0.375
Age (years)	0.97	0.95–0.99	0.005
SOFA without respiration score	1.16	1.06–1.26	<0.001
Septic shock	3.70	1.96–6.99	<0.001
Infection focus (respiratory vs. other), ref = other	1.95	0.96–3.95	0.063
Hypertension	0.70	0.39–1.27	0.245
Diabetes	0.95	0.53–1.72	0.865
Liver disease	1.95	0.77–4.96	0.160
Heart disease	0.53	0.22–1.25	0.147
Cerebrovascular disease	1.00	0.49–2.03	0.999
Lung disease	1.67	0.71–3.95	0.244
Kidney disease	2.05	0.87–4.86	0.102
Malignancy	0.97	0.49–1.93	0.941
Body temperature	0.97	0.76–1.22	0.774
Lactate (mmol/L)	1.11	1.04–1.18	0.002
Procalcitonin (ng/mL)	1.00	0.99–1.01	0.730
White blood cells (× 10^3/μL)	1.00	0.97–1.03	0.960
pH	0.01	0.00–0.03	<0.001
PCO2 (mmHg)	1.04	1.02–1.05	<0.001
HCO3 (mmol/L)	1.00	0.96–1.03	0.899
Glucose (mg/dL)	1.00	0.99–1.01	0.830
CRP (mg/dL)	0.99	0.96–1.02	0.649
Albumin (g/dL)	1.00	0.63–1.60	0.997
High sensitive troponin T (ng/mL)	0.78	0.31–1.95	0.589
BNP elevation	1.11	0.62–2.01	0.721
High-flow nasal cannula use	0.78	0.36–1.67	0.517
ROX index	0.70	0.60–0.80	<0.001
ROX-HR index	0.80	0.72–0.88	<0.001

Abbreviations: CI = confidence interval, ref = reference, SOFA = sequential organ failure assessment, PCO2 = partial pressure of carbon dioxide, HCO3 = bicarbonate, CRP = C-reactive protein, BNP = brain natriuretic peptide, ROX = respiratory rate oxygenation, ROX-HR = ROX-heart rate.
